# Supplementary material for: Social environment mediates cancer progression in Drosophila
Source: Nat Commun. 2018 Sep 3;9:3574. doi: 10.1038/s41467-018-05737-w (PMC6120865; doi:10.1038/s41467-018-05737-w)
Supplement: Supplementary file 1 — Supplementary Information [file 41467_2018_5737_MOESM1_ESM.pdf]

SOCIAL ENVIRONMENT MEDIATES CANCER PROGRESSION IN DROSOPHILA

SUPPLEMENTARY INFORMATION

Dawson et al.

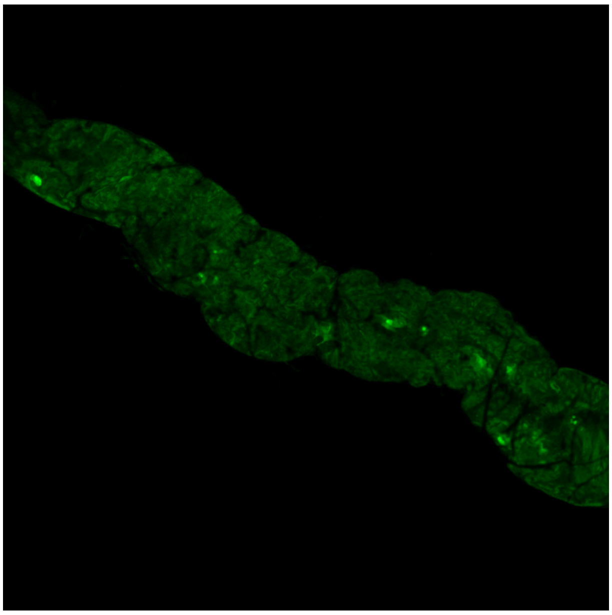

(A) 7 days post induction

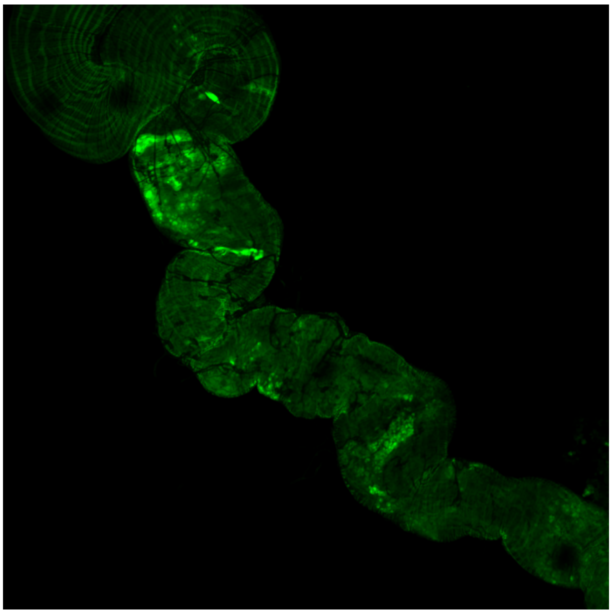

(B) 14 days post induction

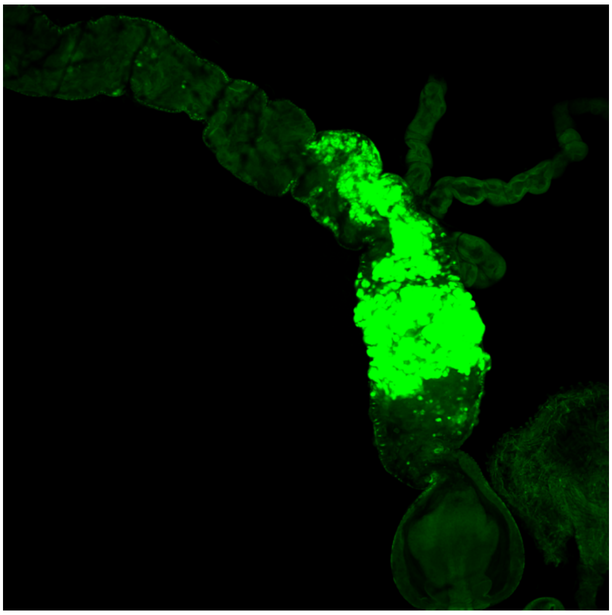

(C) 21 days post induction

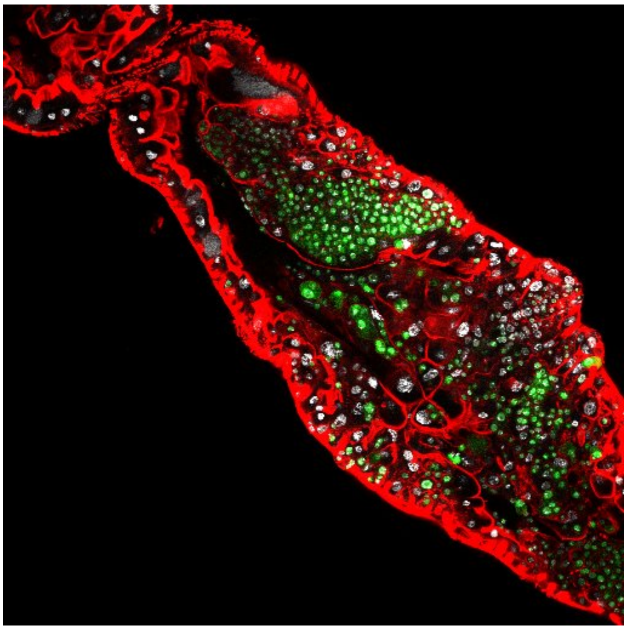

(D) 24 days post induction

Supplementary Figure 1*: Progression of tumor growth in the Midgut.* (**A-C**) APC-Ras clones in the midgut labeled by GFP (green) at 7 days (A), 14 days (B) and 21 days (C) after clonal-induced recombination. Note that 7-day old clones mostly appear as isolated cells, whereas 14-day old clones

appear as groups of few cells; 21-day old clones invade large portions of the midgut. Guts from overnight starved flies were dissected in PBS and fixed with 3,7% formaldehyde in PBT (PBS, 0,1% Tween 20) for 20 mn at room temperature; guts were then extensively washed and mounted in DABCO (sigma). Image acquisitions were obtained using laser scanning confocal microscope (LSM700; Carl Zeiss, Jena, Germany) and a solid-state 488-nm laser for exciting GFP. (**D**) APC-Ras tumoral clone in the anterior midgut labeled by GFP (green) 24 days after clonal-induced recombination. Membrane actin is labeled by Phalloidin (red) and nuclei by DAPI (silver). Note the imbrication of tumoral cells (green) with surrounding control cells (silver).

The A-C images were acquired at a resolution of 1024x1024 pixels using a water immersion objective (x10 achroplan 0.3 NA), whereas the D image was acquired using a Leica SP8 confocal laser-scanning microscope.

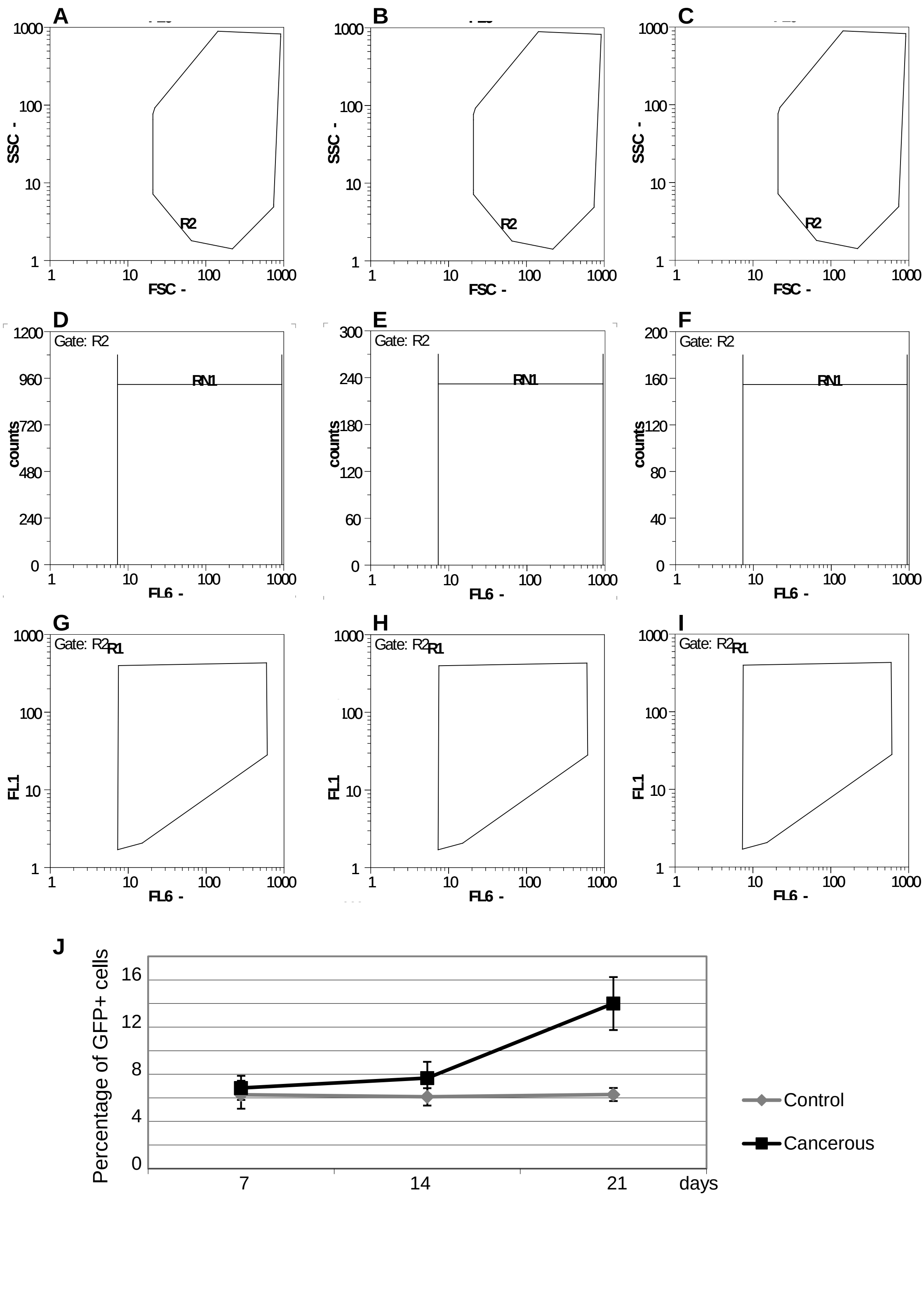

Supplementary Figure 2*: FACS analysis of intestinal cells.* (**A-C**) SSC versus FSC Dotplots to gate (R2) the cells of interest from dissected imaginal discs (A) or guts of w^{1118} (B) and cancerous (C) flies. (**D-F**) Selection of the *Drosophila* cells within the R2 gate, with respect to their DNA content (RN1);

imaginal disc cells allow to identify diploid G1 and G2 cells (2 major pics in D); the intestinal cells of *w*^{1118} (E) and cancerous (F) flies, which undergo DNA endoreplication contain at least the DNA content of G1 imaginal disc cells. (**G-I**) FL6 (DNA) versus FL1 (GFP) Dotplots to select the GFP-positive cells from gut of cancerous flies (R1 in **I**), whereas the R1 gate is empty when analyzing imaginal disc cells (G) and gut cells (H) of *w*^{1118} flies. (**J**) FACS quantification of GFP-positive cells in cancerous (black line) and control (grey line) guts dissected from adult females at 7, 14 and 21 days past HS-induced clonal recombination. Genotype of cancerous and control flies are *HS-flp*;*esg-gal4,UAS-GFP/UAS-Ras*^{V12};*FRT82B,Tub-Gal80/FRT82B,Apc2*^{N175K}*,Apc*^{Q8} and *HS-flp*;*esg-gal4,UAS-GFP*;*FRT82B,Tub-Gal80/FRT82B*, respectively. Error bars: standard error of the mean. N=8 measures for each treatment.

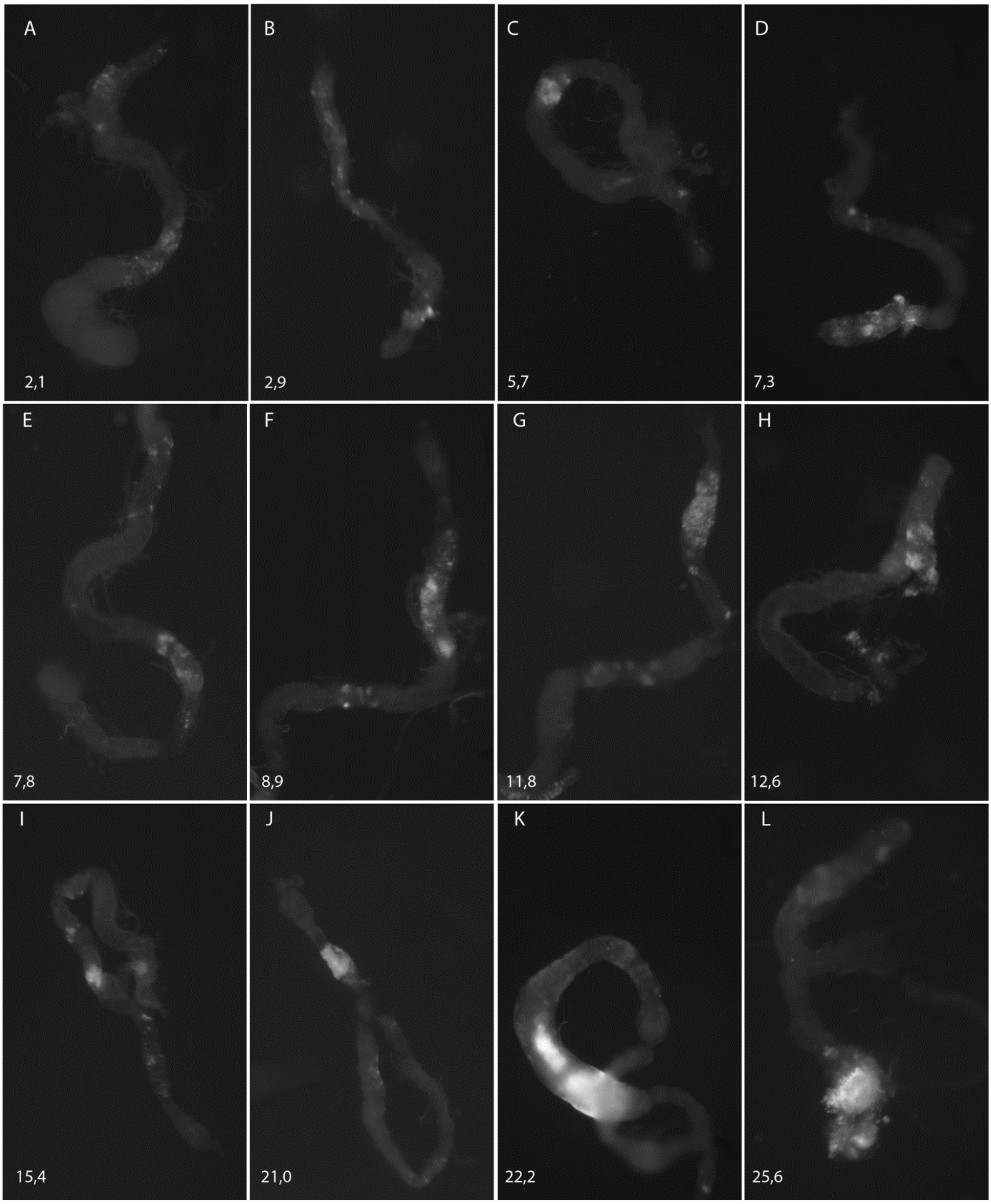
A
2,1
B
2,9
C
5,7
D
7,3
E
7,8
F
8,9
G
11,8
H
12,6
I
15,4
J
21,0
K
22,2
L
25,6

Supplementary Figure 3*: Photos of tumors and corresponding FACS measurements.* . (**A-L**) To evaluate the correlation between the visual appearance of tumors and the associated FACS measurement, twelve guts were dissected and immediately imaged with a dissecting GFP microscope. Subsequently each sample was then individually treated with collagenase, trypsin and Hoechst prior to FACS analysis. The percentage of GFP+ cells, obtained from the FACS analyses, is indicated in the bottom left of each picture. A higher GFP percentage is found for dense tumors, while a lower percentage corresponds to tumors that are intercalated with healthy cells.

Supplementary Note 1

Fly physical performance: We compared physical and behavioral performances between control and cancerous flies at different ages by exposing them to a 'negative geotaxis' behavior test. This involved repeatedly tapping a tube of flies, which causes them to fall, and then measuring the number of individuals that show an escape response (ascending the walls of the tube) over time. When repeatedly performed, this escape response tends to diminish due to exhaustion. This test has been extensively used as a robust proxy to estimate physical and behavioral performance and locomotor activity. We followed a protocol based on the one initially developed by JW Gargano and co-workers [49] and modified by MJ Tinkerhess [50]. Groups of 10 cancerous or control females (7, 14 or 21 days post induction) were introduced into 40ml tubes and placed vertically on a platform which automatically taps the tubes every 4 seconds causing all flies to fall to the bottom of the tube. Every 15 min we visually recorded the number of flies in each tube showing negative geotaxis (climbing at least 2/3 of the tube between two taps). The experiment lasted 2 hours. The data were analyzed with repeated measures ANOVA including time (repeated measure), fly state (cancerous or control) and age (covariate 7,14 or 21 days post induction) as factors. The presence of tumor cells (Apc-Ras clones) has little impact on fly performance and survival over the three weeks of the experimental study. Cancerous and control flies subjected to repeated taps over two hours showed a progressive decline in

negative geotaxis response (Repeated measure ANOVA: time: $F_{1,50}$ = 319,9 $P<10^{-3}$; Fig. S3) which was stronger as flies got older (age: $F_{1,50}$ = 63.1 $P<10^{-3}$; time x age: $F_{1,50}$ = 8.8; P = 0.004). However, no difference between cancerous or control flies could be observed at any age post-induction (state: $F_{1,50}$ = 0.73; P = 0.39; state x age: $F_{1,50}$ = 0.17; P = 0.68) suggesting that despite the general observation of age related impairments, Apc-Ras-induced tumors do not affect physical performance and locomotion 3 weeks after induction.

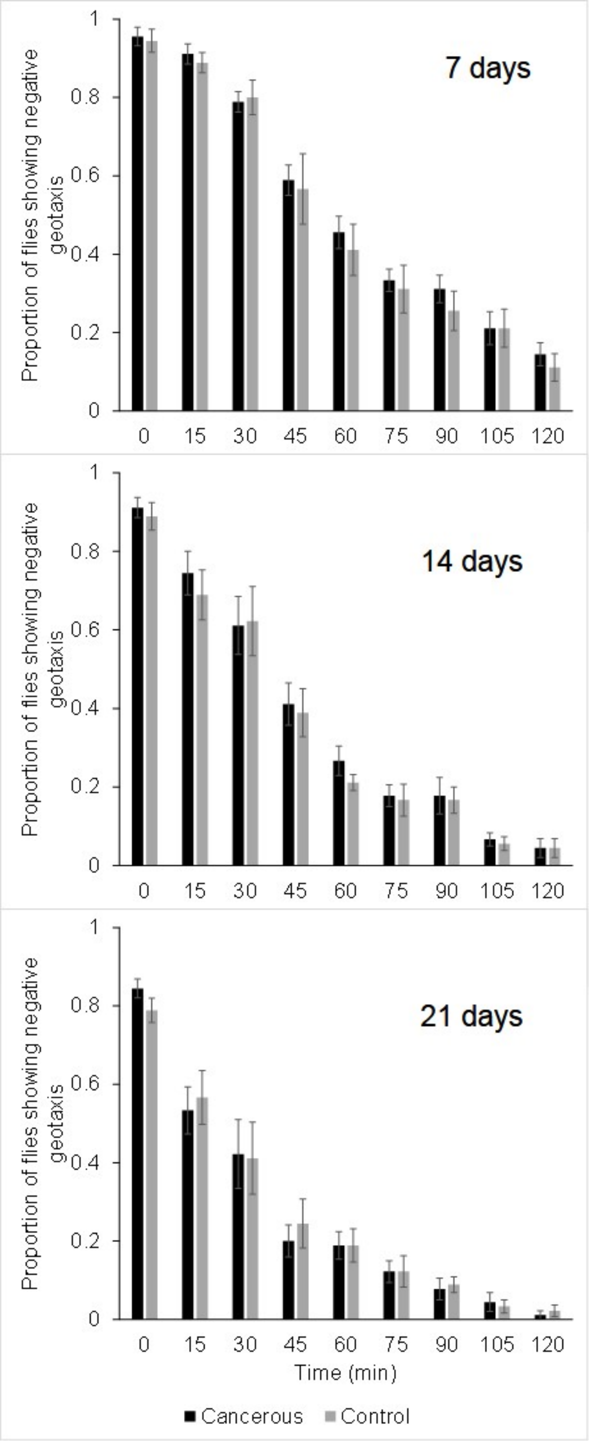

Supplementary Figure 4: *Repeated 'negative geotaxis' behaviour test performed at different ages for cancerous and control flies*. Proportion of flies ascending the walls of the tube after it has been repeatedly tapped. Measures were taken every 15min over 2h at different ages (7, 14 or 21 days post induction). Error bars: standard error of the mean. N=18 groups followed during 2h for each treatment and age**.**
